# Comparative Evaluation of Comprehensive DNA and RNA Sequencing Platforms with Subsequent Clinical Validation for Hematolymphoid Malignancies

**DOI:** 10.3390/cancers18101565

**Published:** 2026-05-12

**Authors:** Julia N. C. Parlow, Nicolas Salcedo-Porras, Fatma AlBulushi, Stephen Yip, Eric McGinnis, Tara Spence

**Affiliations:** 1 Department of Pathology and Laboratory Medicine, Vancouver General Hospital, Vancouver, BC V5Z 1M9, Canadanicolas.salcedoporras@vch.ca (N.S.-P.);; 2 Department of Pathology and Laboratory Medicine, Faculty of Medicine, University of British Columbia, Vancouver, BC V6T 1Z7, Canada

**Keywords:** hematolymphoid malignancies, next-generation sequencing, broad-panel DNA sequencing, RNA exome sequencing, clinical validation, genomic profiling, optical genome mapping, test evaluation

## Abstract

**Background/Objectives:** Genomic alterations play a central role in diagnosis, prognostication, and therapeutic planning for hematolymphoid malignancies. At our tertiary care center, frontline genomic testing relies on optical genome mapping, karyotyping, and fluorescence in situ hybridization, with targeted next-generation sequencing (NGS) performed externally. To provide more comprehensive genomic profiling, we evaluated two large-panel NGS platforms and subsequently performed a clinical validation of the selected assay. **Methods:** The Illumina PanHeme DNA panel and the SOPHiA Genetics Community Myeloid Solution were compared using 24 bone marrow aspirate specimens with previously characterized alterations, including single nucleotide variants (SNVs), insertions/deletions (indels), and copy number variants (CNVs). The selected panel underwent full analytical validation using 60 specimens. **Results:** Both panels demonstrated excellent concordance for SNVs and indels, with comparable analytical performance and workflow. CNV calling with SOPHiA was notably strong. Platform selection was influenced by practical considerations, including panel content and cost, leading to a preference for further evaluation of the Illumina assay. Clinical validation of the Illumina PanHeme DNA panel, along with a complementary RNA Exome panel, was subsequently performed. Sequence variant detection showed 100% concordance with orthogonal testing, while CNV detection was variable, reflecting known limitations of targeted NGS. The RNA panel detected all expected fusion transcripts. **Conclusions:** These findings demonstrate robust analytical performance of both evaluated DNA panels. Clinical validation of the Illumina PanHeme DNA and RNA Exome assays supports their use for comprehensive molecular profiling of hematologic malignancies.

## Simple Summary

Many blood cancers are now diagnosed and managed using detailed genetic testing, but the rapid discovery of new disease-associated genes requires expansion of testing strategies to remain clinically relevant. In our health authority, the current panel for detecting small DNA sequence variants is outdated. We therefore evaluated two modern and expanded DNA sequencing panels to determine their suitability for clinical use, assessing not only analytical performance but also practical workflow considerations relevant to routine diagnostic laboratories. We then performed clinical validation of the selected DNA assay alongside a complementary RNA assay to enable more comprehensive genetic profiling across hematolymphoid malignancies. Both assays demonstrated strong performance, supporting their use in clinical practice. Our findings address a key translational challenge in oncology diagnostics by informing the selection of testing strategies that optimize both analytical performance and clinical utility.

## 1. Introduction

Comprehensive molecular and cytogenetic characterization is essential for accurate diagnosis, risk stratification, and therapeutic management of hematolymphoid malignancies. International diagnostic and risk stratification systems increasingly rely on molecular features to delineate clinical entities, reflecting the expanding role of genomics in precision oncology [1,2,3,4,5,6]. However, no single assay currently captures the full spectrum of clinically relevant genomic alterations, and important gaps remain across the resolution continuum of conventional cytogenetic and molecular methods [7,8,9]. In routine diagnostic workflows, technologies include karyotyping for genome-wide detection of large chromosomal abnormalities, fluorescence in situ hybridization (FISH) for targeted copy number and structural variant assessment, and targeted DNA- and RNA-based PCR or sequencing assays for detection of sequence-level variants and gene fusion events. Genome-wide structural approaches such as optical genome mapping (OGM) additionally provide high-resolution detection of large structural variants, though sequence-based methods are still required to identify smaller sequence-level alterations and other events that fall below the detection threshold of OGM and conventional cytogenetic assays [10]. Together, these modalities occupy distinct but overlapping resolution ranges and are typically integrated in a stepwise or parallel fashion depending on clinical indication, specimen type, and institutional resources.

As precision oncology advances, targeted NGS serves as a critical component of clinical testing workflows due to its ability to sensitively detect clinically actionable single nucleotide variants (SNVs) and small insertions/deletions (indels) and, in some cases, infer exon- or whole-gene copy number variation from the same assay [11,12]. Complementary RNA sequencing can help resolve structural alterations that are ambiguous or not targeted by DNA-based assays—for example, by determining whether splice-site alterations identified in DNA affect transcript processing or by confirming the presence of gene fusions [13,14]. Despite continued improvements in targeted NGS-based copy number and structural variant detection, short-read sequencing remains challenged by complex, repetitive, and intermediate-sized rearrangements [12,15]. Consequently, emerging literature increasingly supports a complementary testing framework in which DNA-based NGS, RNA sequencing, and genome-wide structural assays such as OGM are used together to maximize diagnostic yield for hematologic malignancies [16,17].

Our laboratory, operating within the province’s largest tertiary care facility, provides primary diagnostic services for adult leukemia and bone marrow transplant programs, as well as a large clinical hematology population. The genetic diagnostic workflow currently involves a variable combination of conventional cytogenetics (karyotype), FISH, and OGM depending on the indication for testing, with targeted NGS performed at an external reference laboratory. As the current NGS panel targets regions within 52 myeloid-associated genes and does not broadly include a spectrum of genes relevant to lymphoid malignancies, we sought to evaluate updated NGS panel options suitable for local use that could expand the spectrum of clinically relevant genomic alterations detectable across hematolymphoid malignancies. As an initial step, we performed a comparative evaluation of two vendor-supported DNA-based panels targeting genes relevant to both myeloid and lymphoid malignancies, as well as bone marrow failure and inherited predisposition to hematologic malignancy: Illumina’s Canadian Consortium PanHeme DNA panel (139 genes) and SOPHiA Genetics’ Community Myeloid Solution (111 genes). This assessment was undertaken to inform assay selection based not only on analytical performance but also on practical considerations relevant to deployment in a budget-constrained healthcare system, such as workflow efficiency, turnaround time, cost structure, wet-laboratory complexity, bioinformatic performance, and reporting usability. Both assays employ hybrid-capture enrichment and are compatible with Illumina sequencing instrumentation and flow cells, and each vendor offers complementary RNA-based panels to enable detection of gene fusions and splice-altering events for integrated genomic profiling. At the time of evaluation and publication, the panels represented recently introduced or expanded offerings that had limited real-world clinical experience. Following this evaluation phase, the selected platform was advanced to formal clinical validation in an expanded specimen cohort, and here we report its analytical performance, limit of detection, and reproducibility together with key workflow considerations relevant to clinical implementation.

## 2. Materials and Methods

### 2.1. Cohort for Evaluation of NGS Panels

**Figure 1:**
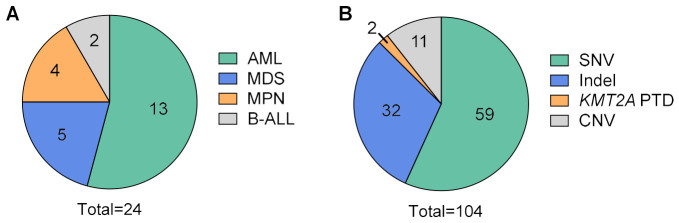
Cohort characteristics for evaluation of Illumina and SOPHiA NGS hematolymphoid panels. (**A**) Specimen distribution by final diagnosis (*n* = 24 specimens). (**B**) Variant categories represented, as determined by orthogonal assays (*n* = 104 variants). The orthogonal assays included targeted next-generation sequencing (SNVs, indels, *KMT2A* PTDs), optical genome mapping (gene fusions, *KMT2A* PTDs, CNVs), and FISH (gene fusions, CNVs). Abbreviations: AML, acute myeloid leukemia; B-ALL, B-cell acute lymphoblastic leukemia/lymphoma; CNV, copy number variant; FISH, fluorescence in situ hybridization; Indel, insertion/deletion/deletion-insertion; MDS, myelodysplastic syndrome; MPN, myeloproliferative neoplasm; PTD, partial tandem duplication; SNV, single nucleotide variant.

Specimens were selected to represent adult hematologic malignancies routinely received for diagnostic testing at Vancouver General Hospital (VGH). Final integrated diagnoses included acute myeloid leukemia (AML, *n* = 13), myelodysplastic neoplasm (MDS, *n* = 5), myeloproliferative neoplasm (MPN, *n* = 4), and B-lymphoblastic leukemia/lymphoma (B-ALL, *n* = 2), as shown in Figure 1A. Across the 24 specimens, 104 clinically relevant DNA variants were identified in 39 unique genes, including 59 SNVs, 32 indels and internal tandem duplications (ITDs), 2 *KMT2A* partial tandem duplications (PTDs), and 11 CNVs (Figure 1B), according to clinical reports from targeted NGS (which can detect SNVs, indels, *KMT2A* PTDs), OGM (which can detect gene fusions, *KMT2A* PTDs, CNVs), and FISH (which detects gene fusions, CNVs) where available. Whereas OGM and FISH were performed in our diagnostic laboratory [7], NGS variants reported by an external clinical laboratory, which uses a custom Illumina (San Diego, CA, USA) DNA sequencing panel targeting 52 genes or hotspots, were retrieved from patient clinical documentation. The *KMT2A* PTDs (14 and 39 kb in size) were analyzed separately from other CNVs because they were the only CNV-type event detectable and reported by the reference NGS panel. All bone marrow aspirate (BMA) specimens were collected in EDTA anticoagulant, frozen within 72 h of collection, and stored at −80 °C until nucleic acid extraction.

### 2.2. DNA Extraction

DNA was extracted from frozen BMA aliquots using the QIAamp DNA Blood Mini Kit following manufacturer recommendations (Qiagen, Hilden, Germany). The resulting DNA concentration was determined by fluorometric quantification using a Qubit 4.0 Fluorimeter (ThermoFisher Scientific, Waltham, MA, USA) with the dsDNA BR kit. All reagent catalog numbers are detailed in Supplementary Table S1. DNA used for reference NGS testing and for the present study was obtained from separate BMA aliquots using different extraction methods, although both aliquots were derived from the same concurrently collected marrow sample.

### 2.3. Evaluation of the Illumina Pan-Hematolymphoid DNA Panel

The Illumina Pan-Hematolymphoid DNA panel (hereafter “PanHeme”) is a newly developed, vendor-supported Canadian custom DNA panel targeting 139 genes relevant in both myeloid and lymphoid malignancies (Illumina). DNA libraries were prepared for NGS using 100 ng of input DNA and following the protocol for Illumina DNA Prep with Exome 2.5 Enrichment v5 with modifications for the Canadian Pan-Heme panel v2 (Illumina), which interrogates 139 genes clinically relevant across a breadth of hematolymphoid malignancies (Supplementary Tables S2 and S3). Quality control of pre-pooled individual libraries included assessment of concentration using the Qubit dsDNA BR Assay Kit with a Qubit fluorometer (ThermoFisher Scientific), and assessment of size using TapeStation D1000 DNA assay reagents with a TapeStation instrument (Agilent, Santa Clara, CA, USA). Final pooled libraries were evaluated using the Qubit dsDNA HS Assay Kit in combination with the TapeStation HS D1000 reagents for the DNA workflow or TapeStation D1000 DNA reagents for the RNA workflow. All reagent catalog numbers are detailed in Supplementary Table S1.

Sequencing was performed on the Illumina NextSeq2000 using P2 flow cells and 300-cycle XLEAP-SBS reagent kits (Illumina). As a sequencing control, PhiX was loaded into the final library to represent 2–4% of total reads, as per manufacturer recommendations. Illumina software was programmed to capture 151 bp paired-end reads and 10 bp index reads, and the unique index sequences were provided to enable on-board demultiplexing and file conversion using DRAGEN 4.2.7 software (Illumina). After sequencing, quality metrics automatically generated by the software were evaluated to ensure that minimal manufacturer-recommended thresholds were met, including ≥90% Q30 score, ≥120 Gigabase yield, ~22 h run time, ≥400 million total single-end reads passing filter, and ≥8 million reads per DNA sample or ≥18 million reads per RNA sample. All reagent catalog numbers are detailed in Supplementary Table S1.

Secondary bioinformatics analysis was performed using the Illumina Connected Analytics (ICA) interface with the DRAGEN analysis pipeline version 4.3.13 with alignment to the GRCh38 genome build (Illumina). Analysis followed the parameters documented in Supplementary Table S4, and the resulting data files were used to assess the concordance between the test method and reference methods. Tertiary analyses including variant annotation were performed using the Illumina Connected Insights (ICI) interface by uploading the secondary data generated in ICA (Illumina).

### 2.4. Evaluation of the SOPHiA Genetics Community Myeloid Solution Panel

The SOPHiA DDM^TM^ Community Myeloid Solution CMYS98_C_v2 (SOPHiA Genetics, Rolle, Switzerland) is a commercially available DNA panel which interrogates 111 genes clinically relevant in hematolymphoid malignancies (Supplementary Table S2). DNA libraries were prepared for NGS using 50 ng of input DNA and following the protocol for SOPHiA GENETICS^TM^ Universal Library Prep v1.3 (SOPHiA Genetics). Quality control steps were performed during and after library preparation, wherein library concentrations were measured using a Qubit fluorometer with the dsDNA HS Assay Kit (ThermoFisher Scientific), and library size was determined using a TapeStation instrument with HS D1000 assay reagents (Agilent). All reagent catalog numbers are detailed in Supplementary Table S1.

Sequencing was performed on the Illumina NextSeq2000 instrument using P2 flow cells and 300-cycle XLEAP-SBS reagent kits (Illumina). As a sequencing control, PhiX was loaded into the final library to represent 2–4% of total reads, as per manufacturer recommendations. Illumina software was programmed to capture 150 bp paired-end reads and 8 bp index reads, and the unique index sequences were provided to enable on-board demultiplexing and file conversion using DRAGEN 4.2.7 software (Illumina). After sequencing, quality metrics automatically generated by the software were evaluated to ensure that minimal thresholds were met, including ≥90% Q30 score, ≥120 Gigabase yield, ~22 h run time, ≥400 million total single-end reads passing filter, and ≥8 million reads per DNA sample. All reagent catalog numbers are detailed in Supplementary Table S1.

Secondary bioinformatics analysis was performed on the SOPHiA Data-Driven Medicine (DDM)^TM^ platform (SOPHiA Genetics) using pipeline ILL1XG1S6_CNV_NextSeq_14 with alignment to the GRCh37 human genome reference, with variant calling optimized for SNVs and short indels and a variant fraction cutoff of 3% for SNVs and indels. The full variant table (FVT) files were used to identify SNVs and indels below 3% VAF as these did not appear on the DDM^TM^ platform.

### 2.5. Panel Comparison—Concordance Analysis

Variants detected by orthogonal assays applied in the routine clinical diagnostic workflow were used as reference or “gold-standard” data sets and variants were selected to facilitate representation of both common but critical and rare clinically relevant variant subtypes, including a range of allelic frequencies and technically challenging loci (e.g., *KMT2A* PTDs, *FLT3* ITDs, and GC-rich targets). Specifically, clinical sequencing performed at an external site was used as reference data for SNVs, indels (including ITDs), and *KMT2A* PTDs; internal lab data from OGM was used as reference for gene fusions, *KMT2A* PTDs, and CNVs; and internal lab data from FISH was used as reference data for gene fusions and CNVs.

Concordance for SNVs and indels across the three NGS panels was assessed using the reference panel as the comparator (assumed ground truth), for which only HGVS cDNA (c.) and protein (p.) nomenclature were available (aligned to the GRCh37 reference genome). For the SOPHiA panel (aligned to the GRCh37 reference genome), concordance was determined by direct comparison of HGVS annotations, requiring exact matches in both c. and p. nomenclature. For the Illumina panel (aligned to the GRCh38 reference genome), variants with matching HGVS annotations were considered concordant; where discrepancies in c./p. annotations were observed, genomic coordinates were compared instead. Specifically, GRCh37 coordinates from the SOPHiA panel were converted to GRCh38 using a lift-over approach (as implemented by the manufacturer), allowing positional equivalence to be confirmed across genome builds. Original and converted coordinates are indicated in Supplementary Table S5.

For CNVs, including *KMT2A* PTDs, concordance was assessed using OGM (GRCh38) as the reference standard. For the Illumina panel (GRCh38), concordance required overlap between reported CNV intervals and the reference regions. For the SOPHiA panel (GRCh37), genomic coordinates were converted to GRCh38 using the Broad Institute LiftOver web tool (https://liftover.broadinstitute.org (accessed on 9 April 2026)), a UCSC-based interface [18], and concordance was defined based on overlap with the reference OGM intervals following coordinate harmonization. Original and converted coordinates are provided in Supplementary Table S5.

For RNA fusions, variants were considered concordant if the same fusion partners were identified. Generally, variants below 5% VAF (DNA) or 5% supporting reads (RNA) were not considered meaningful for assay comparison [19].

### 2.6. Illumina Validation—Cohort

**Figure 2:**
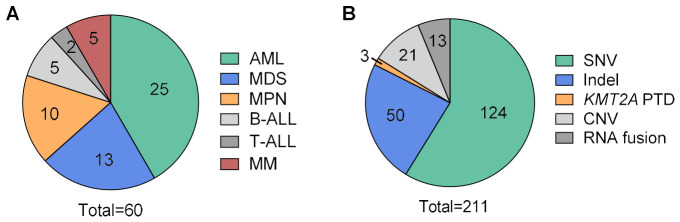
Cohort characteristics for validation of Illumina PanHeme DNA and RNA Exome panels. (**A**) Specimen distribution by diagnosis (*n* = 60 specimens). (**B**) Variant categories represented, as determined by orthogonal assays (*n* = 211 variants). The orthogonal assays included targeted next-generation sequencing (SNVs, indels, *KMT2A* PTDs), optical genome mapping (gene fusions, *KMT2A* PTDs, CNVs), and FISH (gene fusions, CNVs). Abbreviations: AML, acute myeloid leukemia; B-ALL, B-cell acute lymphoblastic leukemia/lymphoma; CNV, copy number variant; FISH, fluorescence in situ hybridization; Indel, insertion/deletion/deletion-insertion; MDS, myelodysplastic syndrome; MPN, myeloproliferative neoplasm; MM, multiple myeloma; PTD, partial tandem duplication; SNV, single nucleotide variant; T-ALL, T-cell acute lymphoblastic leukemia/lymphoma.

Following preliminary assessment of the Illumina PanHeme DNA panel, the cohort of 24 was expanded to include a total of 60 BMA specimens for clinical validation of both the Illumina PanHeme DNA panel and complementary RNA Exome panel, based on guidelines from Jennings et al. [19]. All 60 specimens were processed by both the PanHeme DNA and RNA Exome assays, comprising AML (*n* = 25), MDS (*n* = 13), MPN (*n* = 10), B-ALL (*n* = 5), T-lymphoblastic leukemia/lymphoma (T-ALL, *n* = 2), and multiple myeloma (MM, *n* = 5) as shown in Figure 2A. Across these specimens, orthogonal clinical testing identified 211 clinically relevant DNA and RNA variants, including 124 single nucleotide variants (SNVs), 50 indels (including insertions, deletions, and deletion/insertions), 3 *KMT2A* PTDs, 21 CNVs, and 13 gene fusions (Figure 2B). All BMA specimens were frozen in EDTA anticoagulant within 72 h of collection and stored at −80 °C until nucleic acid extraction (Supplementary Table S6). For the PanHeme DNA panel, 100 ng of input DNA was used (Supplementary Table S7).

### 2.7. Illumina Validation—RNA Exome Workflow

For validation of the Illumina RNA Exome assay, RNA was extracted from frozen BMA using the QIAamp RNA blood Mini Kit with DNase I treatment following manufacturer recommendations (Qiagen). The resulting RNA concentration was determined by fluorometric quantification using a Qubit 4.0 Fluorimeter (ThermoFisher Scientific) with the RNA HS Assay Kit, and the integrity of isolated RNA was assessed using a TapeStation instrument (Agilent) with HS RNA reagents. The input criteria for downstream NGS required that extracted RNA have an integrity score of DV_200_ ≥ 36.5%, where DV_200_ represents the proportion of RNA fragments above 200 nucleotides in length. All reagent catalog numbers are detailed in Supplementary Table S1.

RNA libraries were prepared following the protocol for Illumina RNA Prep with Enrichment (L) Tagmentation v3 (Illumina). Most RNA samples were processed using 50 ng of input, or the maximum available amount when yield was low (Supplementary Table S8). Sequencing followed that of the Illumina PanHeme DNA panel described above, using the same metrics for quality control. Bioinformatic analysis also followed that as described for the Illumina PanHeme DNA panel using the DRAGEN analysis pipeline version 4.3.13 with alignment to the GRCh38 genome build (Illumina), except that default analysis parameters were applied (Supplementary Table S4). All reagent catalog numbers are detailed in Supplementary Table S1.

### 2.8. Illumina Validation—Analytical Performance

Based on concordance analysis (Supplementary Table S9), the analytical performance of the PanHeme DNA and RNA Exome panels (the “test methods”) was evaluated by classifying variants as true positives (TP), false negatives (FN), true negatives (TN), or false positives (FP). For each test method, variants present in the reference datasets (NGS, OGM, and/or FISH) were designated as TP if detected by the test method or FN if not detected. For TN and FP classifications, wherein the absence of variants is assessed, an evaluable space was defined for each variant type, as the absence of all possible variants could not be comprehensively measured. TNs were defined as sequence positions within the evaluable space of reference variants where no variant was detected by either the reference or test method. FPs were defined as positions within the evaluable space where no variant was detected by the reference methods, but a non-polymorphic variant was detected by the test method. For SNVs, the evaluable space encompassed any variant at the coordinates in question. For indels, it included any insertion, deletion, deletion/insertion, or duplication within the relevant coordinates. For CNVs, including *KMT2A* PTDs, it comprised any CNV event occurring within the gene in question. For RNA fusions, it included any fusion partner involving the gene in question. More detailed definitions are provided in Supplementary Table S10. Finally, these designations were used to calculate analytical sensitivity [=TP/(TP + FN)], specificity [=TN/(TN + FP)], and accuracy [=(TP + TN)/(TP + FN + TN + FP)].

### 2.9. Illumina Validation—Linearity and Limit of Detection

Limit of detection (LOD) studies were performed using contrived specimen mixtures generated by reciprocal dilution of clinical samples confirmed to be negative for each other’s target variants based on the validation data obtained (Supplementary Tables S11 and S12). The use of matrix-matched contrived clinical materials has been described in somatic NGS assay validation to approximate native specimen complexity when ideal reference materials are limited [20]. To determine the LOD of SNVs, indels, and fusions, serial dilutions of previously characterized samples were prepared and processed in replicate. Replicate libraries generated at each dilution level were used to assess both LOD and intra-run repeatability (variation between replicates). To accomplish this, for the PanHeme DNA assay, extracted DNA was reciprocally mixed with samples lacking the variants under evaluation to generate preparations at undiluted, 87.5%, 50%, 25%, or 12.5% relative variant abundance, which were then processed in replicates (Supplementary Table S11). For the RNA assay, extracted RNA was similarly mixed at defined proportions to generate preparations with undiluted, 20%, 12.5%, or 5% relative fusion abundance, and libraries were processed in replicates (Supplementary Table S12). Linearity was evaluated by comparing the observed VAF or fusion-supporting read proportion at each dilution to the expected value derived from the reference measurement. The LOD for DNA variants was defined as the expected VAF (based on the reference method) achieved at the lowest dilution level in which the variant remained detectable in all replicates. The LOD for RNA variants was defined as the expected fusion-supporting read proportion (based on the initial run) achieved at the lowest dilution level in which the variant remained detectable in all replicates.

### 2.10. Illumina Validation—Reproducibility

Inter-run and intra-run reproducibility were evaluated for both the PanHeme DNA and RNA Exome assays by re-processing representative specimens from a separate aliquot of extracted nucleic acid (Supplementary Tables S11 and S12). For the DNA assay, four specimens were reprocessed in replicates, and the resulting VAFs were compared between runs and replicates for calculation of inter-run and intra-run reproducibility. Reproducibility was quantified using the coefficient of variation (CV) of VAF measurements, and a CV below 30% was considered acceptable. The RNA assay was evaluated in a similar manner using nine specimens containing fusions with a range of fusion-supporting read counts (a measure of transcript abundance). Fusion-supporting reads (also known as VAF) were defined as:

$$\frac{\left(Alternative\;Paired\;Reads\;+Alternative\;Split\;Reads\right)}{\left(\begin{array}{c} \left(Alternative\;Paired\;Reads\;+Alternative\;Split\;Reads\right)\;\\ +\\ \left(\mathrm{Reference}\;\mathrm{Paired}\;\mathrm{Reads}+\mathrm{Reference}\;\mathrm{Split}\;\mathrm{Reads}\right) \end{array}\right)}$$

## 3. Results

### 3.1. Comparative Performance of Illumina and SOPHiA Genetics Hematolymphoid Panels

The Illumina and SOPHiA Genetics DNA panels were evaluated, using manufacturer protocols without modification, across multiple analytical and operational metrics, including quality control performance, concordance with reference methods, bioinformatic analysis, and reporting usability. The results of these assessments are summarized below.

### 3.2. Quality Control of Illumina and SOPHiA Genetics Hematolymphoid Panels

All 24 specimens passed defined quality control (QC) thresholds during library preparation using both the Illumina PanHeme DNA panel and the SOPHiA Genetics Community Myeloid Solution panel, and all samples achieved greater than eight million sequencing reads (Supplementary Table S5). One specimen containing three previously characterized CNVs (V70) failed analysis on both assays due to excessive CNV noise, possibly related to specimen integrity. This specimen was considered a technical failure and was excluded from all subsequent CNV analyses.

### 3.3. Single Nucleotide Variants

**Figure 3:**
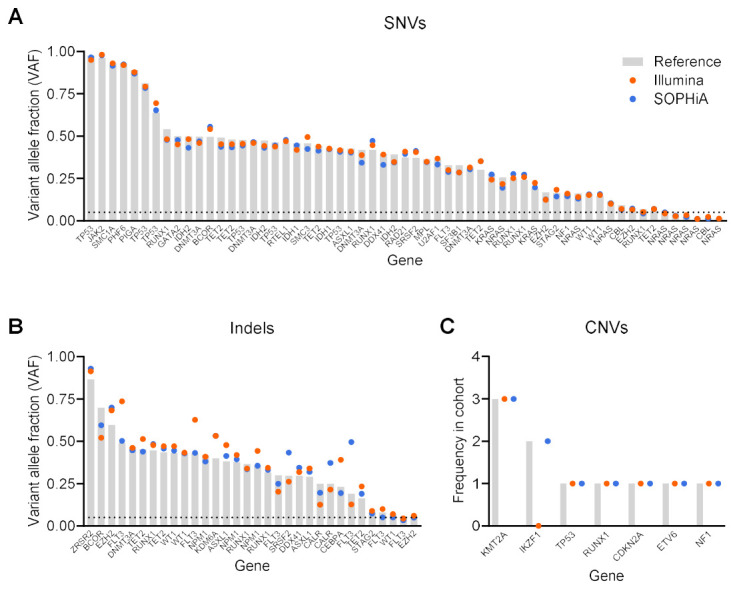
Performance comparison of Illumina PanHeme and SOPHiA Genetics hematolymphoid panels. (**A**) Known SNVs are plotted by VAF as determined by orthogonal clinical testing, with VAFs measured by the Illumina and SOPHiA assays shown for comparison. All SNVs above 5% VAF were reliably detected by both panels. (**B**) Known indels are plotted by VAF, with VAFs measured by the Illumina and SOPHiA assays shown for comparison. All indels were concordantly detected by both methods. (**C**) Known CNVs are plotted according to their frequency in the cohort, and the number of CNVs detected by the Illumina and SOPHiA assays is shown alongside. The Illumina PanHeme panel missed two *IKZF1* deletions, which were detected by the assay upon manual review but were not called by the bioinformatic algorithms. Sample V70 containing 3 known CNVs was excluded from analysis due to high CNV noise in both assays. Dotted line indicates accepted minimal threshold of 5% VAF at diagnosis for oncology testing. Abbreviations: CNV, copy number variant; indel, insertion/deletion/deletion-insertion; SNV, single nucleotide variant; VAF, variant allele frequency.

Across the evaluable specimens, both panels detected all SNVs known by the “gold-standard” reference method, except for a single low-level *NRAS* variant present at 1.4% VAF by the reference method that was not detected by the SOPHiA panel (59/59 or 58/59; Figure 3A). Overall, VAF estimates were similar between the evaluated panels and the reference method: for Illumina, VAF measurements demonstrated < 30% CV across variants, with a mean of 7.0 ± 7.3% CV; one *NRAS* variant showed higher variability (38.3%), consistent with its low abundance (1.9% reference VAF; 1.1% by Illumina). For SOPHiA, all VAF measurements were <30% CV when compared with the reference method, with a mean of 5.8 ± 4.7% CV (Supplementary Table S5). Collectively, both panels showed reliable variant detection down to 2% VAF, with comparable performance for SNV detection and VAF estimation.

### 3.4. Insertions and Deletions

Both panels detected all known indels (32/32; Figure 3B). When compared with the reference method, VAF estimates for indels demonstrated greater variability than for SNVs for both test panels. The mean was 13.7 ± 11.4% CV for Illumina and 11.1 ± 14.0% for SOPHiA (Supplementary Table S5). Variability was particularly high for *FLT3* ITDs, such as the 51bp ITD (49.0% reference VAF; 73.6% by Illumina; 50.3% by SOPHiA) and a 173bp ITD (19.0% reference VAF; 12.8% by Illumina; 50.0% by SOPHiA). Increased variability was also observed for technically challenging GC-rich variants, including a *CALR* indel (25.0% reference VAF; 21.6% by Illumina; 37.3% by SOPHiA) and a *CEBPA* indel (23.1% reference VAF; 39.1% by Illumina; 19.5% by SOPHiA).

### 3.5. Copy Number Variants

Both CNVs detected by reference NGS (2/2 *KMT2A* PTDs) were concordantly identified by both the Illumina and SOPHiA assays. We additionally evaluated eight CNVs identified by OGM, which varied in genomic size and gene coverage, including amplification of *KMT2A*, and deletions involving *IKZF1*, *TP53*, *RUNX1*, *CDKN2A*, *ETV6*, *NF1*. Of these, six were concordantly detected by both assays based on statistically significant CNV calls within the expected gene and overlap of call coordinates with the reference CNV intervals (Figure 3C, Supplementary Table S5). However, two *IKZF1* deletions in the Illumina workflow were detected but did not pass quality filtering thresholds and were therefore considered missed.

### 3.6. Library Preparation Workflow

The Illumina PanHeme and SOPHiA Community Myeloid workflows follow broadly similar library preparation principles, including DNA fragmentation, PCR amplification, library QC and pooling, hybridization-based target capture, post-capture amplification, and final QC (Table 1). Overall hands-on workflow duration was comparable between platforms, but total assay time was slightly higher for Illumina in our experience (total time ~11.5 h for Illumina, ~10 h for SOPHiA). Although the SOPHiA protocol includes a longer initial fragmentation step due to longer incubations, the Illumina workflow utilizes additional bead clean-up, washing, and reagent thawing steps. Specifically, the Illumina protocol includes both double-sided and single-sided bead clean-ups with ethanol washes throughout the protocol, whereas the SOPHiA workflow relies primarily on ethanol wash steps.

The Illumina documentation provides highly detailed stepwise instructions, including reagent preparation, estimated step durations, and multiple optional pause points (with validated storage at −20 °C for up to 30 days). In contrast, the SOPHiA protocol is more streamlined but less prescriptive (for example indicating optional storage at 4 °C overnight or −20 °C for “longer”). In practice, our laboratory adapts vendor protocols into internally standardized operating procedures where we may adjust the protocol as desired.

### 3.7. Input Requirements and Pooling

Recommended DNA input requirements are similar between platforms (Table 1): SOPHiA recommends 50 ng input (acceptable range 10–100 ng with PCR cycle adjustment), whereas Illumina recommends ≥ 50 ng (acceptable range 10–1000 ng with PCR cycle adjustment). Maximum input volume is higher for SOPHiA (40 µL) compared with Illumina (30 µL). Sample pooling capacity is comparable but with slightly greater flexibility with Illumina (up to 12 samples per pool for Illumina, or 4, 8, or 12 samples for SOPHiA). Recommended final library loading concentrations are identical (1000 pM).

### 3.8. Equipment and Laboratory Workflow Considerations

The SOPHiA workflow utilizes 8-tube strips, whereas the Illumina protocol uses 96-well plates (Table 1). Tube-strip handling requires repeated capping and uncapping while the plate format requires a compatible plate shaker and plate-compatible centrifuge rotors but may improve batch handling efficiency. The SOPHiA workflow additionally requires a vacuum concentrator, and both methods require distinct heat blocks and magnetic separation devices. Hybridization setup differs in complexity. The SOPHiA protocol requires reagent addition within a thermocycler maintained at 65 °C and staggered timing when processing batches greater than 8, and it benefits from two post-PCR thermocyclers (whereas Illumina requires one). The Illumina hybridization-capture steps may be operationally simpler; however, both workflows are amenable to automation, which may mitigate manual workflow differences in high-throughput laboratories with liquid handler capabilities.

### 3.9. Assay Performance and Informatics

All samples in the evaluation cohort passed quality control requirements with both platforms. Both analysis pipelines provide comparable core features, including variant summaries, QC metrics, oncogenicity predictions, population and internal frequency data, curated annotations, and links to external knowledge bases. The Illumina variant interpretation software ICI v5.2.1 provides more extensively integrated clinical evidence from external databases such as CKB, OncoKB, and CIViC, while the SOPHiA variant interpretation software DDM offers an opt-in, community-derived variant knowledge sharing feature across participating laboratories known as “Collective Intelligence” as well as a particularly user-friendly analytical interface (Table 1). According to vendor-reported documentation, ICI relies on the DRAGEN analysis suite, utilizing proprietary AI-driven tools like SpliceAI and PrimateAI-3D to enhance variant prioritization and oncogenicity predictions, while the SOPHiA platform incorporates proprietary algorithms such as MUSKAT™ for adaptive CNV calling and PEPPER™ for signal-to-noise differentiation, designed to address technical challenges in complex genomic regions, and is sequencer agnostic.

A distinguishing feature of the SOPHiA ecosystem is the associated MaxCare services, which provide optional vendor-supported analytical workflows and formalized validation support. Through these services, SOPHiA Genetics can assist laboratories with assay validation by performing data analysis, concordance assessment, and calculation of performance metrics. This approach may be particularly advantageous for laboratories with limited dedicated bioinformatics or validation resources, where validation activities must be performed alongside routine clinical workloads. However, both vendors provided excellent technical and informatics support throughout the evaluation process, including responsive field support, sales engagement, and assistance with workflow integration. Both platforms were adaptable to laboratory needs and provided timely troubleshooting and guidance during assay evaluation.

Based on the analytical performance observed in this study, both assays represent strong options for clinical testing in hematologic malignancies. Ultimately, platform selection was influenced by several practical considerations, including panel content and cost. The Illumina panel included several genes of clinical interest to our practice, and the overall assay cost was modestly lower. In the context of a resource-constrained healthcare system, these factors supported selection of the Illumina assay for clinical implementation at our center.

### 3.10. Clinical Validation of Illumina DNA and RNA Assays

#### 3.10.1. Quality Control

**Figure 4:**
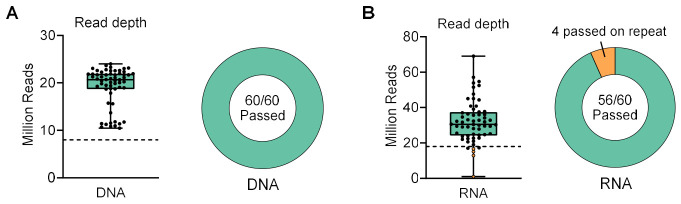
Sequencing read depth achieved upon first attempt for Illumina DNA and RNA samples. (**A**) The target read depth for DNA was ≥8 million reads (dotted line). (**B**) The target read depth for RNA was ≥18 million reads (dotted line). Four RNA samples were re-processed to achieve sufficient read depth. Two RNA samples achieved just below 18 million reads but were considered passed.

Based on the comparative evaluation of Illumina and SOPHiA DNA panels, the Illumina PanHeme DNA panel was selected for subsequent clinical validation. We also validated the Illumina RNA Exome panel in parallel, as a complementary test to identify critical gene fusions and the impact of splice-site variants in hematologic malignancies. The initial cohort of 24 was expanded to include a total of 60 BMA specimens. DNA and RNA were extracted from the same specimens, and all 60 DNA samples (Supplementary Table S7) and 60 RNA samples (Supplementary Table S8) passed library preparation QC checkpoints upon first attempt. After sequencing, all 60 DNA samples had achieved the target read depth (≥8 million reads) on first attempt (Figure 4A). For RNA, 56 of 60 samples achieved the manufacturer-recommended threshold of ≥18 million reads on first attempt, and the 4 samples which failed were re-processed and re-sequenced, achieving sufficient read depth upon a second attempt either independently or by merging data with the first attempt (Figure 4B).

#### 3.10.2. Variant Concordance

**Figure 5:**
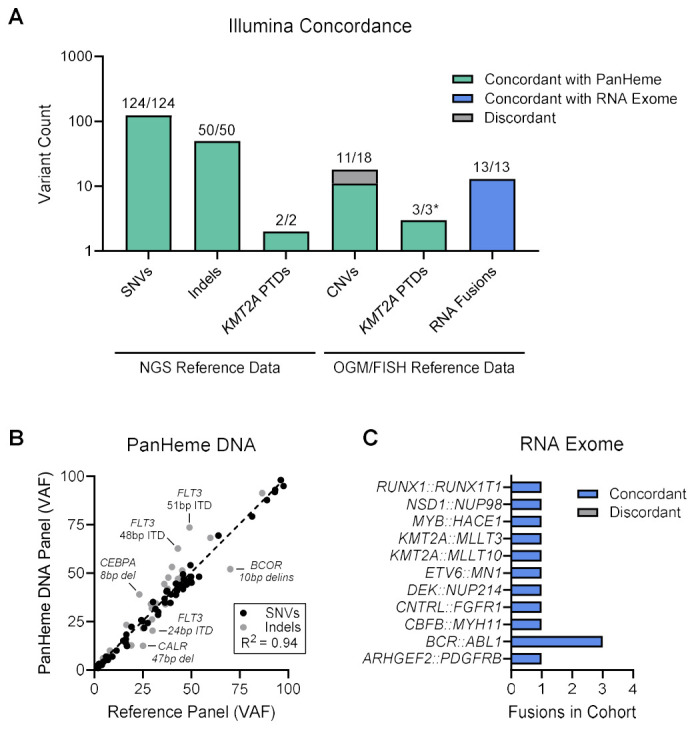
Concordance of Illumina PanHeme DNA and RNA Exome panels with reference datasets. (**A**) DNA results were compared to clinical sequencing, OGM, and FISH, while RNA results were compared to OGM and/or FISH. * Two of the *KMT2A* PTDs detected by OGM were also accounted for by the NGS reference dataset and are therefore counted twice. Note that sample V70 failed CNV quality and was excluded. (**B**) Correlation of observed VAFs with reference VAFs determined by orthogonal clinical sequencing for SNVs and indels. (**C**) Concordant and discordant RNA fusion variants. Abbreviations: FISH, fluorescence in situ hybridization; indel, insertion/deletion/deletion-insertion; NGS, next generation sequencing; OGM, optical genome mapping; PTD, partial tandem duplication; SNV, single nucleotide variant; VAF, variant allele frequency.

The expected variants previously detected by NGS, OGM, and/or FISH were compared to the findings by Illumina PanHeme DNA and RNA Exome assays to determine concordance (Supplementary Table S9). Concordance for sequence variants was defined as the detection of variants previously identified by orthogonal NGS testing. All sequence-level variants (100%; 174/174 variants) detected by clinical NGS were concordantly detected by the PanHeme DNA panel, including 124 SNVs, 50 indels, and 2 *KMT2A* PTDs (Figure 5A).

Copy number alterations were evaluated separately. Except for *KMT2A* PTDs, the remaining CNVs included in the comparison set were previously identified by OGM rather than orthogonal NGS. Within this context, all *KMT2A* PTDs were detected (3/3), whereas the rate of detection of CNVs in other genes was lower (11/18). Discordant findings included four *IKZF1* deletions that were either not detected or failed variant filtering, and one *CDKN2A* deletion that did not pass filtering criteria. Two low-frequency *TP53* deletions (estimated VAF ~2% and 4%), previously detected in enriched plasma cells were not identified.

The observed VAF from SNVs and indels by the PanHeme DNA panel correlated strongly with the expected VAF reference values (Figure 5B), with the exception of variants occurring in genomic contexts known to challenge accurate quantification, including larger *FLT3* ITDs, where allele fraction estimation is more complex because the duplication may span multiple sequencing reads [21], and variants in GC-rich regions of *CEBPA* [22].

The RNA Exome panel correctly identified a range of fusion variants (13/13) reflected in the DNA by OGM and/or FISH (Figure 5C). Assessment of global RNA expression was outside the scope of the current evaluation; the RNA panel was used primarily to confirm putative gene fusions identified by structural methods and to support interpretation of splice-altering variants detected by DNA sequencing.

#### 3.10.3. Analytical Performance

The analytical performance of the Illumina PanHeme DNA and RNA Exome panels was evaluated by defining variants as true positives, false negatives, true negatives, or false positives (Supplementary Table S10). These values were then used to calculate analytical sensitivity, specificity, and accuracy (Table 2). Overall performance was excellent, with all sequence-level variants previously detected by orthogonal clinical NGS concordantly identified by the PanHeme DNA panel, including 124 SNVs, 50 indels, and 3 *KMT2A* PTDs, corresponding to 100% sensitivity for sequence variants. Performance of copy number alterations differed; five of the 16 expected CNVs were missed, including four *IKZF1* deletions and one *CDKN2A* deletion, all of which were detected but did not pass quality filtering.

#### 3.10.4. Linearity and Limit of Detection

**Figure 6:**
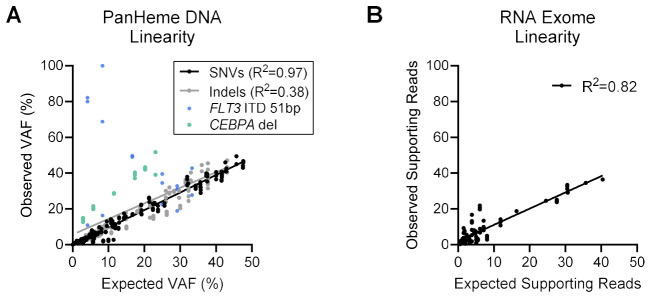
Linearity of Illumina NGS panels. (**A**) Dilution curves using undiluted, 87.5%, 50%, 25%, or 12.5% variant abundance DNA samples were prepared in replicates and processed by the PanHeme DNA panel. Linearity was assessed based on the degree of closeness between observed and expected values, where expected VAF was calculated based on the reference dataset. Outliers occurred for the *CEBPA* 8bp deletion and the 51bp *FLT3* ITD, as previously seen. (**B**) Dilution curves using undiluted, 20%, 12.5%, or 5% relative fusion abundance RNA samples were prepared in replicates and processed by the RNA Exome panel. Linearity was assessed based on the degree of closeness between observed and expected values, where expected fusion-supporting reads were calculated based on the initial run. Abbreviations: Indel, insertion/deletion/deletion-insertion; SNV, single nucleotide variant; VAF, variant allele fraction.

Linearity and LOD were evaluated using dilution series generated from contrived mixtures of clinical specimens, with replicate libraries used to assess both parameters (Supplementary Tables S11 and S12). Both assays demonstrated linearity in the dilution series, with observed values closely matching those expected/calculated from the reference values (Figure 6). Taken together, linearity performance was strong for both DNA and RNA, with outliers occurring in regions known to be challenging to call informatically (the GC-rich gene *CEBPA* and the *FLT3* 51bp ITD).

Based on dilution performance, SNVs were confidently detected at 0.40–4.63% VAF, and indels were confidently detected at 1.00–4.95% VAF (Table 3, Supplementary Table S11). The LOD for CNVs was not assessed due to variability in analytical performance for CNV detection. Overall, conservative LODs were established at 5% VAF for both SNVs and indels [19].

Detection of fusions was assessed across dilutions, and detection was successful for most cases down to a 5% abundance of the original sample, which corresponds to 5% fusion-supporting reads (Table 3, Supplementary Table S12). For the intended application of fusion detection, in which the transcript frequency for such fusions tends to be high (≥10%) at the time of diagnosis, the demonstrated ability of the assay to reliably detect fusions in substantially diluted specimens indicates adequate performance for clinical deployment. Therefore, the LOD was established at 10% fusion-supporting reads (corresponding to approximately eight reads supporting the fusion event). An extensive LOD evaluation of RNA LOD was deemed out of scope for the purpose of this validation assessment, as available “gold-standard” reference values were not available.

#### 3.10.5. Reproducibility

**Figure 7:**
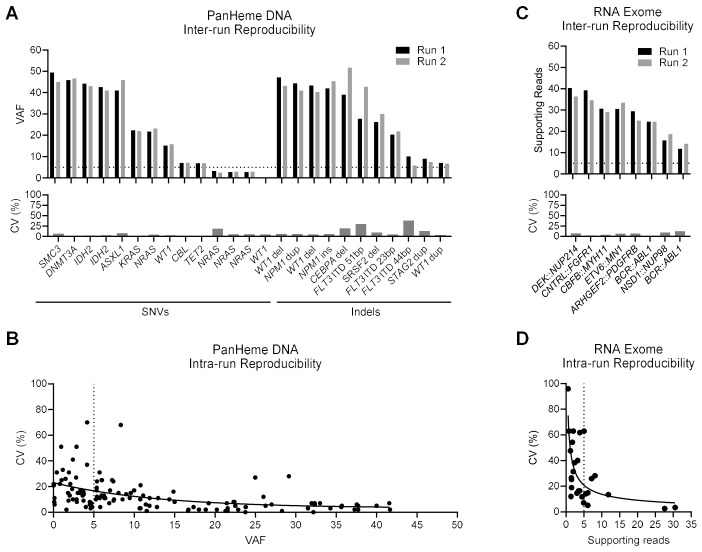
Inter- and intra-run reproducibility of Illumina PanHeme DNA and RNA Exome assays. (**A**) DNA specimens containing a variety of representative variants were re-processed through library preparation and sequencing to evaluate inter-run reproducibility, and the %CV of the VAF between runs is shown for variants in loci indicated. (**B**) Intra-run reproducibility for DNA samples was assessed across assay replicates, with the %CV of observed VAF plotted against the expected VAF. (**C**) RNA specimens containing a variety of representative fusions were re-processed through library preparation and sequencing to evaluate inter-run reproducibility, and the number of fusion-supporting reads is shown, as well as the %CV between runs. (**D**) Intra-run reproducibility for RNA samples was assessed across assay replicates, with the %CV of observed supporting reads plotted against the expected number of supporting reads. Dotted line indicates accepted minimal threshold of 5% VAF (or supporting reads) at diagnosis for oncology testing. Abbreviations: CV, coefficient of variation; indel, insertion/deletion/deletion-insertion; SNV, single nucleotide variant; VAF, variant allele fraction.

For the Illumina PanHeme DNA assay, reproducibility was evaluated using four specimens from the validation cohort (run 1) which contained representative SNVs and indels at a range of VAFs. Library preparation and sequencing was replicated (run 2) using DNA derived from a separate aliquot of extracted nucleic acid. Comparison of run 1 and 2 showed strong inter-assay reproducibility of VAF values, with most variants showing a CV below 20%, except for regions known to be challenging to sequence or call such as larger *FLT3* ITDs and the GC-rich regions of *CEBPA* (Figure 7A, Supplementary Table S11). Intra-assay reproducibility was also strong for the PanHeme DNA panel, with less than 20% CV generally observed amongst replicates, except for variants with low VAF where small differences in VAF inflate the CV (Figure 7B, Supplementary Table S11). The *FLT3* 51bp ITD remained an outlier, likely because its size falls near the boundary between variant callers used for detection of small versus large indels.

Inter-run and intra-run reproducibility of the RNA Exome panel was assessed similarly, using nine specimens from the validation cohort which contained fusions with a range of fusion-supporting reads (a measure of transcript abundance) determined in run 1. Library preparation and sequencing was replicated (run 2) using RNA derived from a separate aliquot of extracted nucleic acid. Inter-assay reproducibility was strong, with less than 10% CV generally observed between runs (Figure 7C, Supplementary Table S12). Intra-assay reproducibility was variable for low-abundance (diluted; see section below) variants but was strong for higher-abundance (undiluted) variants, which better represent the abundance expected in diagnostic specimens (Figure 7D, Supplementary Table S12).

## 4. Discussion

### 4.1. Clinical Significance

As molecular classifications continue to expand the number of genomic alterations that carry diagnostic, prognostic, and therapeutic significance, clinical laboratories require testing strategies that can detect diverse variant classes within clinically meaningful timeframes [23]. Targeted NGS panels have become an essential component of modern diagnostic workflows, enabling sensitive detection of sequence-level variants while complementing structural genomic methods used in hematologic malignancy testing [24]. Moreover, RNA sequencing provides additional clinically relevant information by enabling direct detection of fusion transcripts and supporting the interpretation of splice-altering variants that may be difficult to resolve using DNA-based assays alone [13,14]. In this study, we aimed to evaluate two targeted DNA sequencing panels and subsequently validate an integrated DNA and RNA sequencing approach to support the molecular profiling of hematolymphoid malignancies within our diagnostic laboratory.

### 4.2. Evaluation and Comparison of Illumina and SOPHiA Hematolymphoid Panels

During the evaluation and comparison of two targeted NGS panels, the Illumina PanHeme DNA panel and SOPHiA Community Myeloid panel demonstrated successful library generation across all analyzed specimens and excellent concordance with reference methods. Variability in VAF was greater for low-abundance variants, larger indels, and challenging genomic alterations such as *FLT3* ITDs and *CEBPA* variants, which reflect the known limitations of NGS [21,22]. Both panels successfully detected *KMT2A* PTDs, but detection of CNVs orthogonally detected by OGM was variable—while the SOPHiA assay detected 10/10 CNVs; two *IKZF1* deletions did not pass quality filtering thresholds by Illumina and were therefore considered missed.

Differences in CNV detection performance may reflect underlying methodological differences in normalization and variant-calling approaches for CNVs. According to vendor-reported workflows, the SOPHiA Genetics assay relies on internal normalization across samples within a sequencing run, therefore requiring at least eight samples be processed per batch, whereas the Illumina workflow uses an external panel-of-normals (PoN) reference framework, in addition to distinct proprietary CNV calling algorithms. These methodological differences may contribute to the stronger CNV performance observed with the SOPHiA panel. This conclusion should be interpreted cautiously as the assessment involved only 24 specimens, but it may be relevant for laboratories where CNV detection is a priority. Importantly, different orthogonal reference methods were used across variant classes (NGS for SNVs/indels and OGM/FISH for CNVs and structural variants), which may influence the interpretation of concordance across variant types due to inherent differences in resolution, genomic coverage, and detection principles. CNV detection ultimately depends on event size, tumor cell fraction, breakpoint architecture, and methodological differences such as probe design (FISH) or computational approach [25], which could limit the sensitivity of targeted NGS compared with FISH and OGM. Consequently, these methods are best viewed as complementary, supporting an integrated testing framework in which DNA-based NGS, RNA sequencing, and OGM are used together to maximize diagnostic yield for hematologic malignancies [16,17].

From a workflow perspective, both the Illumina and SOPHiA assays demonstrated broadly comparable overall processing time, supporting their suitability for clinical deployment. The Illumina workflow was slightly more technically prescriptive and incorporates additional clean-up steps, which are designed to promote library consistency and provide highly integrated downstream annotation, albeit with somewhat greater procedural complexity and hands-on burden. In contrast, the SOPHiA protocol was slightly more streamlined and may simplify initial laboratory implementation, with differences between platforms largely reflecting alternative workflow designs rather than fundamental technical limitations. Informatically, both platforms provide robust variant interpretation environments with strong knowledge base support, and both vendors demonstrated responsive technical support and provided implementation resources that facilitated assay onboarding in our laboratory, with the SOPHiA platform being particularly user-friendly and supportive in facilitating clinical validation design and analysis. Collectively, these observations indicate that both solutions represent viable options for clinical laboratories, and that platform selection depends on local priorities such as workflow preferences, current infrastructure, panel design, cost structure, CNV calling capabilities, and degree of bioinformatic support desired. In this context, cost considerations are highly context-dependent and may vary substantially between institutions due to vendor-specific pricing agreements, procurement timing, testing volumes, and bundling of reagents, instrumentation, or service contracts; accordingly, cost observations from a single center may not be broadly generalizable. In our setting, the Illumina panel was selected for implementation based on cost and the number and composition of genes targeted. However, both vendors may introduce expanded or updated panel designs in the future, which could alter the comparative cost and feature landscape.

### 4.3. Clinical Validation of the Illumina PanHeme DNA and RNA Exome Panels

During clinical validation of the Illumina NGS panels, first-pass success rates were 100% with the DNA panel and 93% with the RNA panel, with all RNA specimens passing read depth minimums upon a second attempt. RNA read depth failures were attributed primarily to sample quality, likely reflecting the availability and use of frozen specimens for RNA extraction rather than fresh material. Variability in RNA integrity may have introduced bias during probe capture in pooled libraries, preferentially enriching higher-quality samples and causing large variability in the sequencing read depth achieved. To mitigate this effect, subsequent libraries were pooled according to RNA input quality, with samples exhibiting lower DV_200_ values grouped together, in accordance with the manufacturer’s recommendations. Following this adjustment, repeat sequencing runs met quality control criteria for nearly all specimens, with only two samples remaining marginally below the target threshold of 18 million reads.

The DNA panel showed excellent sensitivity for sequence-level variants, with all previously identified SNVs and indels detected in the validation cohort. Variability in VAF between reference results and the validation cohort may also be influenced by specimen source, as DNA was extracted from separate BMA aliquots using distinct DNA extraction techniques, though derived from a marrow specimen collected concurrently. Sensitivity for CNVs was lower, reflecting factors discussed earlier, as well as the use of conservative variant filtering thresholds that are intended to minimize false-positive calls. Evaluation of this variant class was limited by the relatively small number of CNVs included in the cohort, reflecting both our preliminary observations during assay evaluation which led to de-prioritization of CNV analysis and the limited availability of established orthogonal methods within the existing diagnostic workflow. The RNA Exome panel detected all expected fusion transcripts in the validation cohort, supporting the value of RNA sequencing as a complementary approach to DNA-based testing by OGM. Assessment of gene expression was outside the scope of the present evaluation, as the RNA panel was primarily used for fusion confirmation and splice variant interpretation within the diagnostic workflow. However, RNA expression analysis may later be evaluated due to its reported usefulness in confirming the presence of novel fusion transcripts and aberrant gene expression [13].

Analytical validation metrics further supported the suitability of the Illumina assays for clinical implementation, demonstrating 100% specificity, sensitivity, and accuracy for SNVs, indels, and *KMT2A* PTDs. A conservative limit of detection of 5% VAF was established for both SNVs and indels, consistent with clinical validation guidelines for targeted NGS panels at diagnosis [19], though variants were detectable at lower allele fractions. For fusion detection, a reporting threshold of 10% fusion-supporting reads was established, indicating that 10% of the reads spanning the region in question corresponded to the alternative fusion transcript. In practice, this threshold generally corresponded to an absolute count of at least eight reads supporting the fusion event. Many RNA-based LOD studies report the dilution factor at which a fusion remains detectable; however, this metric does not directly convey the absolute abundance of the fusion transcript [26,27]. Linearity analysis demonstrated a strong correlation between observed and expected values for both the DNA and RNA panels, except for the 51 bp *FLT3* ITD and the *CEBPA* variant which were consistently outliers due to the challenging nature of these variants. Reproducibility was also strong, with coefficients of variation below 20% for most variants evaluated.

LOD and reproducibility analyses were performed using contrived dilution series derived from clinical specimens due to the limited availability of well-characterized reference materials, reflecting the practical realities of many clinical laboratories operating within publicly funded healthcare systems. Within this framework, conservative reporting thresholds were selected to ensure reliable assay performance in clinical testing. We anticipate that this pragmatic validation approach may serve as a useful reference for other laboratories implementing similar assays under comparable resource constraints.

## 5. Conclusions

Combined genomic testing using targeted DNA and RNA sequencing enables detection of multiple clinically relevant variant classes, including SNVs, indels, CNVs, and gene fusions, thereby supporting more comprehensive diagnostic characterization of hematologic malignancies. In this study, both the Illumina and SOPHiA targeted DNA panels demonstrated strong analytical performance in the comparative evaluation cohort, indicating that either platform represents a suitable option for clinical implementation. Ultimately, platform selection may depend on laboratory-specific priorities such as gene content, cost structure, bioinformatic infrastructure, and extent of validation support required. Subsequent clinical validation of the Illumina PanHeme DNA and RNA Exome panels further demonstrated excellent performance for SNVs, indels, *KMT2A* PTDs, and gene fusions, supporting their use as an integrated platform for routine molecular profiling in hematologic malignancy diagnostics.

## Figures and Tables

**Table 1:** Workflow comparison of evaluated platforms.

Parameter	Illumina PanHeme	SOPHiA Community Myeloid
Workflow	Day 1 (*~5.5* h): Tagmentation Post-tagmentation clean-up PCR amplification Post-amplification clean-up QC check Pooling Hybridization overnight Day 2 (*~6* h): Reagent thaw (2 h) Capture PCR amplification Post-amplification clean-up QC check *Total estimated time: 11.5* h	Day 1 (*~7* h): Fragmentation, ligation Post-ligation clean-up PCR amplification Post-amplification clean-up QC check Pooling and concentration Hybridization overnight Day 2 (*~3* h): Capture PCR amplification Post-amplification clean-up QC check *Total estimated time: 10* h
Input	Amount of DNA: 10–100 ng (≥50 ng recommended) Maximum volume: 30 µL	Amount of DNA: 10–100 ng (50 ng recommended) Maximum volume: 40 µL
Pooling Capacity	1 to 12 samples (commonly 12)	4, 8, or 12 samples
Equipment differences	Utilizes 96-well plates Magnetic stand for 96-well plates Heat block for 96-well plates High speed microplate shaker Microplate centrifuge	Utilizes 8-tube strips Magnetic stand for 8-tube strips Heat block for 1.5 mL tubes Vacuum concentrator Two post-PCR thermal cyclers ideal
Secondary Bioinformatics	Illumina Connected Analytics (ICA) with DRAGEN analysis	SOPHiA DDM^TM^ Platform
Tertiary Bioinformatics	Illumina Connected Insights (ICI) -*More integration of clinical evidence*	SOPHiA DDM^TM^ Platform -*Particularly user-friendly* -*Formalized validation support*

Italics indicate subjective information.

**Table 2:** Analytical performance of NGS panels.

Parameter *	PanHeme DNA				RNA Exome
	SNVs (*n* = 124)	Indels (*n* = 50)	*KMT2A*-PTDs (*n* = 3)	CNVs ** (*n* = 16)	Fusions (*n* = 13)
Sensitivity (%)	100.00	100.00	100.00	68.75	100.00
Specificity (%)	100.00	100.00	100.00	100.00	100.00
Accuracy (%)	100.00	100.00	100.00	98.92	100.00

* Two myeloma specimens were excluded from all analyses due to low plasma cell percentages. ** Sample V70 was excluded from CNV analysis due to high CNV noise. Abbreviations: SNV, single nucleotide variant; Indel, insertion/deletion/deletion-insertion; PTD, partial tandem duplication; CNV, copy number variant.

**Table 3:** Lower limit of detection of Illumina NGS panels.

Parameter	PanHeme DNA		RNA Exome
	SNV (*n* = 14)	Indel (*n* = 11)	Fusion (*n* = 8)
Analytical LOD	0.40–4.63% VAF	1.00–4.95% VAF	1–8% fusion-supporting reads
Reported LOD	≥5%	≥5%	≥10% fusion-supporting reads

Abbreviations: SNV, single nucleotide variant; Indel, insertion/deletion/deletion-insertion; LOD, limit of detection.

## Data Availability

The original contributions presented in this study are included in the article/Supplementary Material. Further inquiries can be directed to the corresponding author.

## Supplementary Materials

The following supporting information can be downloaded at https://www.mdpi.com/article/10.3390/cancers18101565/s1: Table S1: Catalog Numbers; Table S2: Panel Gene Lists; Table S3: Illumina PanHeme Targets; Table S4: Illumina Bioinformatics; Table S5: Panel Comparison; Table S6: Validation Cohort; Table S7: DNA QC; Table S8: RNA QC; Table S9: Variant Concordance; Table S10: Analytical Performance; Table S11: DNA LOD and Reproducibility; Table S12: RNA LOD and Reproducibility.
